# Xanthocillin X Dimethyl Ether Exhibits Anti-Proliferative Effect on Triple-Negative Breast Cancer by Depletion of Mitochondrial Heme

**DOI:** 10.3390/md23040146

**Published:** 2025-03-28

**Authors:** Jingjing Du, Xuening Zhang, Kaiqiang Guo, Wanjun Lin, Wenjian Lan, Zi Wang, Meina Shi, Zifeng Huang, Houjin Li, Wenzhe Ma

**Affiliations:** 1 School of Pharmacy, Faculty of Medicine & State Key Laboratory of Quality Research in Chinese Medicine, Macau University of Science and Technology, Macau 999078, China; jingjing6sun@163.com (J.D.); 3220000038@student.must.edu.mo (X.Z.); guokq@scu.edu.cn (K.G.); wjlin@must.edu.mo (W.L.); 2009853ucw30009@student.must.edu.mo (Z.W.); 21098537ct30001@student.must.edu.mo (M.S.); 3220005672@student.must.edu.mo (Z.H.); 2 School of Pharmaceutical Sciences, Sun Yat-Sen University, Guangzhou 510006, China; lanwj@mail.sysu.edu.cn; 3 School of Chemistry, Sun Yat-Sen University, Guangzhou 510006, China

**Keywords:** xanthocillin X dimethyl ether, triple-negative breast cancer, heme, mitochondria

## Abstract

Triple-negative breast cancer (TNBC) presents a significant therapeutic challenge due to the absence of specific targeted treatments. In this study, we explored the therapeutic potential of xanthocillin X dimethyl ether (XanDME), a naturally occurring isocyanide isolated from the marine fungus *Scedosporium apiospermum*, on TNBC. To elucidate the underlying mechanism, we initially demonstrated that XanDME directly binds to hemin, the oxidized form of heme, in vitro, corroborating previous reports. This interaction led to the depletion of intracellular regulatory heme. We further established that XanDME translocates into the mitochondria, where it interacts with crucial hemoproteins, namely cytochromes. The binding of XanDME with mitochondrial cytochromes disrupts the electron transport chain (ETC), inhibits the activity of mitochondrial complexes, and inactivates mitochondrial respiration. The inhibitory activity of XanDME on mitochondrial function significantly contributes to its anti-TNBC effects, as observed both in vitro and in vivo. Our study underscores the potential of XanDME against TNBC, warranting further investigations.

## 1. Introduction

Triple-negative breast cancer (TNBC), accounting for 12% of all breast cancer cases, poses significant therapeutic challenges due to its heterogeneity and the limited availability of specific targeted treatments [1]. Consequently, understanding vulnerabilities and identifying targeted molecules are crucial components of TNBC management. Altered metabolic processes, a hallmark of cancer, have garnered significant attention in the development of anti-cancer agents. Recent research has demonstrated that certain types of cancer exhibit functional or even enhanced mitochondrial respiration, contrary to the canonical Warburg hypothesis [2]. Therefore, targeting the mitochondrial electron transport chain (ETC) has emerged as an attractive approach for cancer treatment, including TNBC, which features elevated mitochondrial oxidative phosphorylation (OXPHOS) capacity [3,4].

Heme, also known as iron protoporphyrin IX, is a tetrapyrrole molecule that contains iron and plays an essential role in various biological processes. It achieves this by integrating into different hemoproteins, including globins, mitochondrial cytochromes, and cytochrome P450 enzymes [5]. Among these proteins, mitochondrial cytochromes assume vital significance in the ETC, facilitating the transfer of electrons between specific complexes. Depletion of the cytochrome complex (Cytc) results in mitochondrial dysfunction [6]. Interestingly, certain cancer cells, characterized by heightened OXPHOS capacity, exhibit elevated levels of heme and heme synthesis [7,8,9]. Conversely, heme deficiency impairs mitochondrial respiration and restrains cancer growth and metastases [10,11]. Therefore, chelating heme as a means to induce mitochondrial dysfunction emerges as a promising therapeutic strategy for cancer treatment, particularly in the context of TNBC.

**Figure 1:**
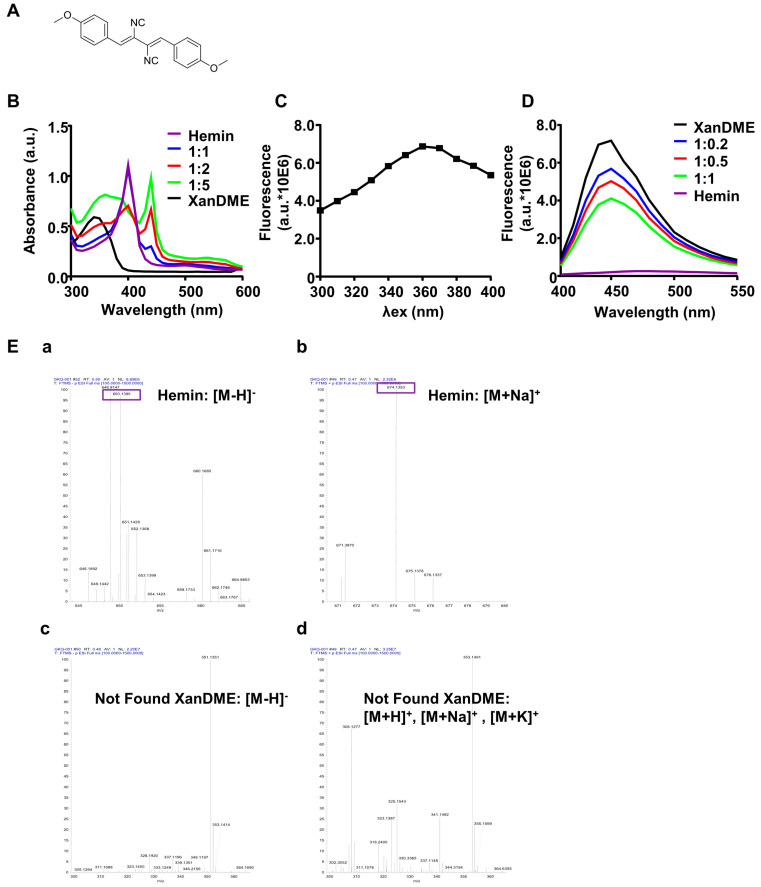
In vitro binding of XanDME to hemin. (**A**) Depiction of XanDME’s molecular structure. (**B**) UV−Vis spectrum of hemin (20 μM), XanDME (20 μM), and different proportions of hemin: XanDME. (**C**) Fluorescence spectrum of XanDME, with emission wavelength set at 450 nm. (**D**) Fluorescence spectrum of XanDME (10 μM), hemin (10 μM), and different proportions of XanDME: hemin. (**E**) High-resolution mass spectra of 1:1 mixture of XanDME and hemin. (**a**) Negative ion mode spectra with [M−H]^−^ peak of hemin highlighted. (**b**) Positive ion mode spectra with [M+Na]^+^ peak of hemin highlighted. (**c**) Negative and (**d**) Positive ion mode spectra with mass range 300–365 for XanDME ions.

Xanthocillin X dimethyl ether (XanDME, Figure 1A), a derivate of xanthocillin X (Xan), was initially isolated and identified from the mycelium of the soil fungus *Aspergillus* sp. [12]. Subsequently, it has demonstrated a range of pharmacological activities against viruses, bacteria, thrombocytopenia, and Alzheimer’s disease [13,14,15,16]. Moreover, XanDME exhibits inhibitory effects on the proliferation of multiple cancer cell lines, including those derived from hepatoma, breast cancer, and epithelial carcinoma [17]. Recently, our group isolated XanDME from a marine fungus *Scedosporium apiospermum* and confirmed its anti-proliferative activity [18]. Marine-derived compounds have emerged as significant sources of novel antitumor agents, with numerous studies demonstrating their potential in cancer therapy [19]. The unique chemical structures and diverse biological activities of these marine natural products offer promising avenues for developing effective anticancer drugs [20]. Recent research has particularly highlighted the contributions of marine fungi, which produce a variety of bioactive secondary metabolites with potent antitumor effects [21]. These compounds, including alkaloids, polyketides, and terpenoids, have shown remarkable efficacy in inhibiting cancer cell proliferation, inducing apoptosis, and overcoming drug resistance in various cancer models [21]. The exploration and utilization of marine-derived compounds, especially those from marine fungi, represent a crucial direction in the ongoing quest for more effective and less toxic cancer treatments.

XanDME contains two isonitrile groups, which have been proposed to bind to heme [22,23]. Recently, experimental evidence has confirmed the binding of XanDME with heme [15]. Consequently, we hypothesized that the interaction between XanDME and heme may contribute to its anti-proliferative activities. In this study, we demonstrate that XanDME directly binds to hemin in vitro. This interaction results in the depletion of intracellular regulatory heme, leading to mitochondrial dysfunction and ultimately inhibiting the proliferation of TNBC cells and tumor growth.

## 2. Results

### 2.1. XanDME Binds to Heme In Vitro

It has been reported that Xan and its derivatives possess the ability to bind with hemin, the oxidized form of heme, thereby altering its ultraviolet–visible (UV–Vis) spectral characteristics [15]. In line with these findings, hemin exhibited an absorption peak at 390 nm (Figure 1B). The addition of XanDME resulted in a significant transformation of the UV–Vis absorption spectrum of hemin, exhibiting a peak shift to 440 nm with the increased absorption in a dose-dependent manner (Figure 1B). Xan is recognized as an autofluorescence molecule, with an optimal excitation wavelength at 366 nm and emission at 414 nm [15]. To ascertain if XanDME also exhibits autofluorescence and to determine its spectral profiles, an initial scan of the excitation spectrum of XanDME was conducted, revealing an excitation maximum at 360 nm (Figure 1C), consistent with its absorption peak (Figure 1B). The emission spectrum of XanDME was subsequently identified, with a maximum at 450 nm (Figure 1D). The addition of hemin led to a dose-dependent decrease in fluorescence intensity (Figure 1D). Collectively, the observed alterations in the absorbance and emission spectra suggest a direct interaction between hemin and XanDME. This interaction was further confirmed by high-resolution mass spectrometry (HRMS) analysis of a mixture of XanDME and hemin. The analysis detected the ions of hemin ([M−H]^−^: 650.1389, [M+Na]^+^: 674.135), but none of the ions of XanDME were found (Figure 1E).

### 2.2. Biochemical Inactivation and Cellular Depletion of Heme upon XanDME Binding

**Figure 2:**
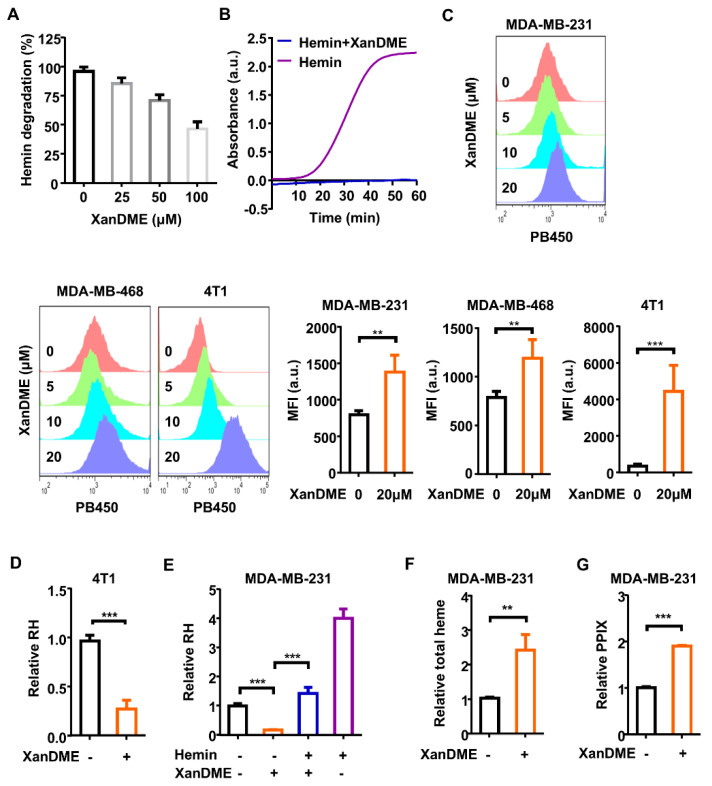
The biochemical inactivation and cellular depletion of heme upon XanDME binding. (**A**) Dose-dependent inhibition of the GSH-mediated degradation of hemin (25 μM) by XanDME. (**B**) In vitro peroxidase activity assay conducted in the presence of hemin, with or without XanDME. Hemin (5 nM) was preincubated with either XanDME (25 μM) (blue) or assay buffer (purple). The time dependent response of the hemin-promoted peroxidase activity was represented by the absorption (λem = 570 nm) in kinetic mode. (**C**) Flow cytometry measurements of the intracellular XanDME in TNBC cells (MDA-MB-468, MDA-MB-231, 4T1) post 24 h treatment at 5, 10, 20 μM using the PB450 channel. The data represent the average values ± SD of independent experiments (n = 3 per group). (**D**) The regulatory heme (RH) content in 4T1 cells measured by hemin colorimetric assay post 48 h treatment with XanDME (5 μM). (**E**) The RH content in MDA-MB-231 cells by hemin colorimetric assay. Cells were treated with XanDME (5 μM) for 48 h, with or without a 2 h pretreatment of hemin (10 μM). (**F**) The total heme content in MDA-MB-231 cells measured by fluorescence (λem = 610 nm, 660 nm) post 24 h treatment with XanDME (5 μM). (**G**) Porphyrin (PPIX) content in cell lysate measured by fluorescence (λex = 406 nm). MDA-MB-231 cells were incubated with either XanDME (5 µM) or DMSO for 24 h, following a 2 h pretreatment with ALA (0.1 mM) (** *p* < 0.01; *** *p* < 0.001).

What are the implications of XanDME binding to heme? It is known that heme undergoes degradation in vitro in the presence of glutathione (GSH) [24]. Preincubation with XanDME prevented GSH-mediated hemin decomposition in a dose-dependent manner (Figure 2A). This suggests that the binding of XanDME leads to the biochemical inactivation of heme. This was further demonstrated by an in vitro peroxidase activity assay. Hemin, acting as a cofactor, triggered the peroxidase reaction, leading to an increase in the product absorbed at 570 nm. However, preincubating hemin with XanDME fully blocked the reaction (Figure 2B).

Taking advantage of the autofluorescent properties of XanDME, we were able to quantify its intracellular levels by flow cytometry. As expected, a significant increase in the intracellular fluorescence of XanDME was observed in two human TNBC cell lines, MDA-MB-468 and MDA-MB-231, as well as in the mouse TNBC cell line 4T1, following an incubation period of 24 h (Figure 2C). Regulatory heme (RH), a form of heme responsible for the regulation of hemoproteins, encompasses free heme, weak protein-binding heme, and newly synthesized heme [25]. The reduction in the RH level induced by XanDME was found in 4T1 cells (Figure 2D). This phenomenon was significantly replicated in MDA-MB-231 cells, and pretreatment with hemin effectively restored the RH levels (Figure 2E). Interestingly, the total intracellular heme level significantly increased following XanDME treatment (Figure 2F). This could potentially indicate a compensatory increase in heme biosynthesis, as evidenced by the elevated level of protoporphyrin IX (PPIX), a precursor of heme (Figure 2G).

### 2.3. XanDME Induces Mitochondrial Dysfunction

**Figure 3:**
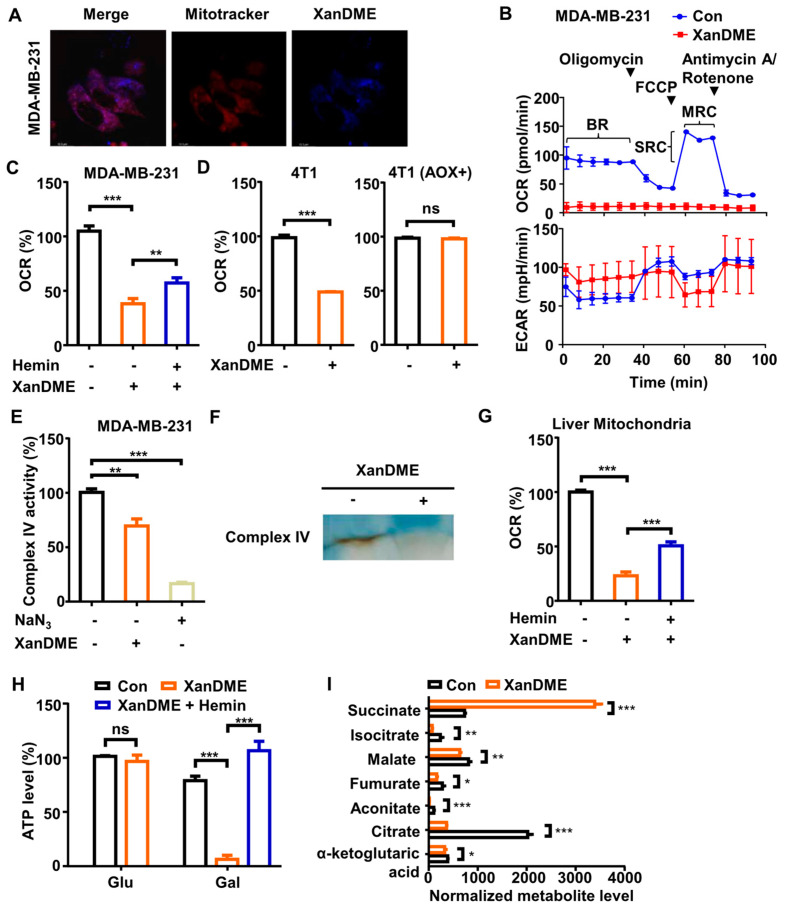
XanDME induces mitochondrial dysfunction. (**A**) TNBC cells were treated with XanDME (20 µM) for 24 h. Subsequently, the mitochondria were labeled with Mitotracker Deep Red. The colocalization of XanDME (blue) and mitochondria (red) was analyzed using fluorescence imaging (scale bar, 12.3 μm). (**B**) The oxygen consumption rate (OCR) and the extracellular acidification rate (ECAR) of MDA-MB-231 cells were measured using an XFp Analyzer following treatment with XanDME (5 μM) for 48 h. (**C**) The OCR of MDA-MB-231 cells were recorded using the Instech Oxygen Consumption Measurement system after treatment with XanDME (5 μM) for 24 h and pretreatment with hemin (10 μM) for 2 h. The OCR was normalized by cell number and calculated relative to the control group. (**D**) The OCR of 4T1 cells and 4T1 (AOX+) cells were measured following treatment with XanDME (5 μM) for 24 h. The OCR was normalized by cell number and calculated relative to the control group. (**E**) The mitochondrial complex IV activity of MDA-MB-231 cells after treatment with XanDME (5 μM) for 24 h. (**F**) The mitochondrial complex IV activity of isolated liver mitochondria after incubation with XanDME (100 μM) for 5 m by an in-gel activity assay. (**G**) The OCR of isolated liver mitochondria after pretreatment with XanDME (30 μM), with or without hemin (30 μM) for 30 m. (**H**) The ATP content of MDA-MB-231 cells grown in glucose or galactose medium after treatment with XanDME (5 μM) for 48 h, with or without pretreatment with hemin (10 μM) for 2 h. (**I**) The metabolites of MDA-MB-231 cells in the TCA cycle were detected by metabolomics analyses after treatment with XanDME (5 μM) for 48 h (* *p* < 0.05; ** *p* < 0.01; *** *p* < 0.001; ns, *p* > 0.05).

Heme, serving as a critical cofactor of various hemoproteins, plays a significant role, particularly in relation to the ETC components located within the inner mitochondrial membrane. This is due to the presence of heme in respiratory complexes II, III and IV, as well as in the electron carrier cytochrome c. The autofluorescence of XanDME enables its visualization using a confocal fluorescent microscope. It showed that a considerable fraction of XanDME colocalized with Mitotracker in MDA-MB-231 cells (Figure 3A). To explore the potential impact of XanDME on mitochondrial function, we initially utilized the Seahorse Bioscience extracellular flux (XF) analyzer to examine the metabolic profile of MDA-MB-231 cells treated with XanDME. It is noteworthy that the oxygen consumption rate (OCR) of cells treated with XanDME was remarkably diminished, while the control group maintained the normal basal respiration (BR), maximal respiratory capacity (MRC), and spare respiratory capacity (SRC). (Figure 3B). Meanwhile, the extracellular acidification rate (ECAR) exhibited a relative increase in cells treated with XanDME (Figure 3B). This suggests a compensatory metabolic adaptation and eliminates the possibility that the decrease in OCR was a result of cell death. The influence of XanDME on cellular OCR was validated using a Clark-type oxygen microelectrode. The inhibition was significantly alleviated by pretreatment with hemin (Figure 3C). Alternative oxidase (AOX), a peroxidase from sea squirt, can bypass mitochondrial complexes III and IV, directly converting O_2_ into water [26]. Consequently, the expression of AOX in 4T1 cells counteracted the OCR inhibition by XanDME (Figure 3D). This implies that the effects of XanDME on mitochondrial function are mediated by complexes III and/or IV.

To analyze the direct effects of XanDME, mitochondria were isolated from MDA-MB-231 cells, and the activity of complex IV was measured using an in-gel assay. This approach was chosen because a recent study demonstrated a selective effect on complex IV, rather than on complexes II or III, when heme was depleted [27]. It showed that XanDME treatment significantly decreased complex IV activity with NaN_3_ as a positive control (Figure 3E). This was verified in isolated mouse liver mitochondria using the spectrophotometric assay (Figure 3F). Similar to the results observed in cells (Figure 3B,C), the OCR measured in isolated mouse liver mitochondria decreased significantly with XanDME treatment, and this effect was partially reversed by pretreatment with hemin (Figure 3G).

To further elucidate the metabolic effects of XanDME, we measured cellular ATP levels in MDA-MB-231 cells post-XanDME administration. Surprisingly, no significant disparity was observed between the ATP levels of XanDME-treated cells and those of control cells when cultured in a glucose medium (Figure 3H). This is likely attributable to the compensatory increase in ECAR (Figure 3B). However, a notable reduction in cellular ATP levels was observed when the cells were cultured in a medium where glucose was replaced with galactose (Figure 3H). Given the slower metabolism of galactose via glycolysis, cells cultured in a galactose medium predominantly rely on OXPHOS for ATP production [28]. Consequently, the diminished ATP concentration in XanDME-treated cells substantiates its impact on mitochondrial functionality. Moreover, pre-incubation with hemin effectively restored the cellular ATP concentration (Figure 3H).

Furthermore, inhibition of the ETC has been linked to dysregulated metabolites in the tricarboxylic acid cycle (TCA cycle) [29]. Therefore, we characterized the effects of XanDME on TCA cycle metabolites using liquid chromatography–mass spectrometry (LC–MS). In line with previous findings, a majority of the TCA cycle metabolites, including α-ketoglutaric acid, citrate, aconitate, fumarate, malate, and isocitrate, exhibited a decrease upon XanDME treatment (Figure 3I). However, succinate levels significantly increased following XanDME treatment (Figure 3I). This is likely due to the presence of a crucial hemoprotein, cytochrome b, located in the succinate dehydrogenase, also known as respiratory complex II, which facilitates the conversion of succinate to fumarate in the TCA cycle. The binding of XanDME to cytochrome b results in the inactivation of the enzyme complex, thereby leading to an accumulation of the substrate succinate.

### 2.4. XanDME Selectively Inhibits Cell Proliferation in TNBC Cells

**Figure 4:**
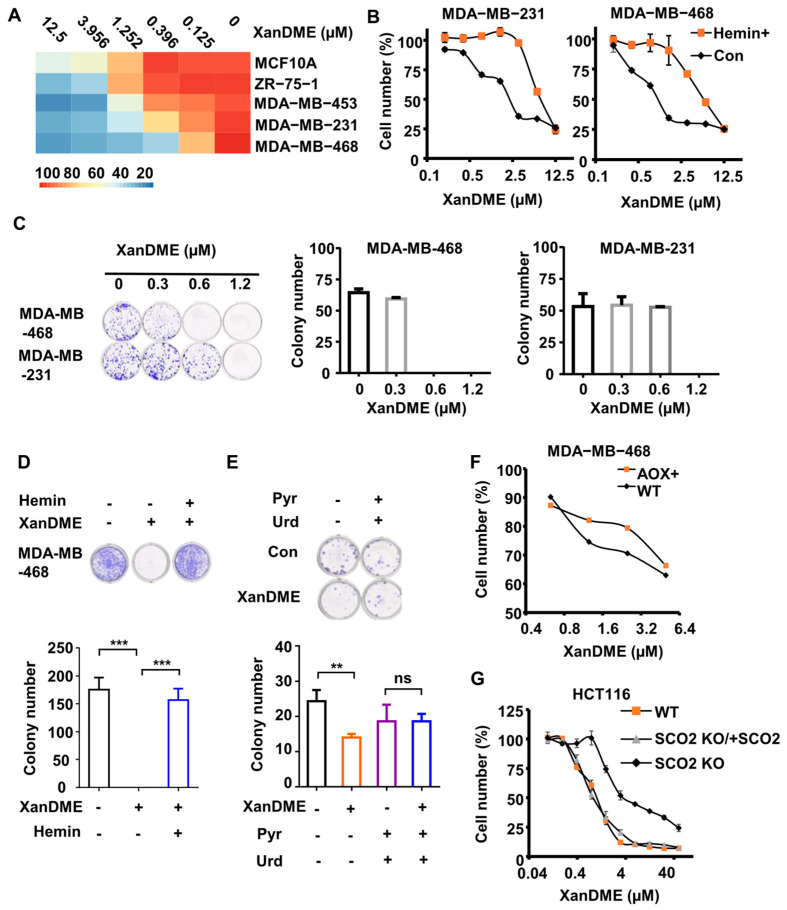
XanDME selectively inhibits cell proliferation in TNBC cells. (**A**) The number of various cells following 72 h treatment with XanDME at a series of concentrations. (**B**) The number of MDA-MB-231 cells and MDA-MB-468 cells following 72 h treatment with XanDME at a series of concentrations, with or without a 2 h pretreatment with hemin (10 μM). (**C**) Colony formation in MDA-MB-231 and MDA-MB-468 cells following 10 d treatment with XanDME. (**D**) Colony formation in MDA-MB-468 cells following 10 d treatment with XanDME (5 μM), with or without a 2 h pretreatment with hemin (10 μM). (**E**) Colony formation in MDA-MB-231 cells following 10 d treatment with XanDME (0.625 μM), with or without a 2 h pretreatment with pyruvate (1 mM) and uridine (50 μg/mL). (** *p* < 0.01; *** *p* < 0.001; ns, *p* > 0.05) (**F**) The number of 4T1 (WT), 4T1 (AOX+), MDA-MB-468 (WT), and MDA-MB-468 (AOX+) cells following 72 h treatment with XanDME at a series of concentrations. (**G**) The number of HCT116 (WT), HCT116 (SCO_2_ KO), HCT116 (SCO_2_ KO/+SCO_2_) cells following 72 h treatment with XanDME at a series of concentrations.

Blockages in the ETC have been associated with a decrease in cell proliferation [30]. Consequently, we evaluated the impact of XanDME on cell proliferation across different types of breast cancer cell lines. XanDME demonstrated an inhibition of cell proliferation on all tested breast cancer cell lines in a dose-dependent manner. Interestingly, the half-maximal inhibitory concentration (IC_50_) values for TNBC cell lines MDA-MB-231 and MDA-MB-468 cells were 0.85 μM and 0.25 μM, respectively (Figure 4A). TNBC cell lines were more sensitive to XanDME than ZR-75-1 (ER+, PR+/−, HER2−) and MDA-MB-453 (ER−, PR−, HER2+) cells, whose IC_50_ values were 1.7 μM and 1.1 μM, respectively (Figure 4A). Furthermore, the IC_50_ value of XanDME treatment in the normal breast cell line, MCF10A, was determined at 5.5 μM (Figure 4A). These findings suggest that XanDME selectivity targets TNBC cells, which align with their vulnerability to mitochondrial inhibitors. Moreover, pretreatment with hemin significantly attenuated the XanDME-induced inhibition of cell proliferation in MDA-MB-231 and MDA-MB-468 cells (Figure 4B). Additionally, XanDME significantly inhibited colony formation in MDA-MB-231 and MDA-MB-468 cells in a dose-dependent manner (Figure 4C). Remarkably, the addition of hemin almost completely restored colony formation in MDA-MB-468 cells treated with XanDME (Figure 4D).

Cells exhibiting a dysfunctional ETC are auxotrophic for pyruvate and uridine [31]. Consequently, the addition of pyruvate and uridine into the culture medium resulted in the recovery of colony formation in MDA-MB-231 cells (Figure 4E) compared to cells treated solely XanDME. Similarly, the ectopic expression of AOX to reconstruct the ETC in XanDME-treated MDA-MB-468 cells significantly enhanced their tolerance to the compound (Figure 4F). Finally, the inhibitory effect of XanDME on cell proliferation, contingent on the mitochondrial ETC, was verified using HCT116 SCO2 KO cells. These cells are derived from the human colon cancer HCT116 cells, characterized by the knockout of the cytochrome c oxidase 2 synthesis gene. Given their deficiency in respiration [32], HCT116 SCO_2_ KO cells exhibited relative resistance to XanDME (Figure 4G). Moreover, the re-expression of SCO_2_ in these cells reinstalled their sensitivity to XanDME (Figure 4G).

### 2.5. XanDME Inhibits TNBC Tumor Growth

**Figure 5:**
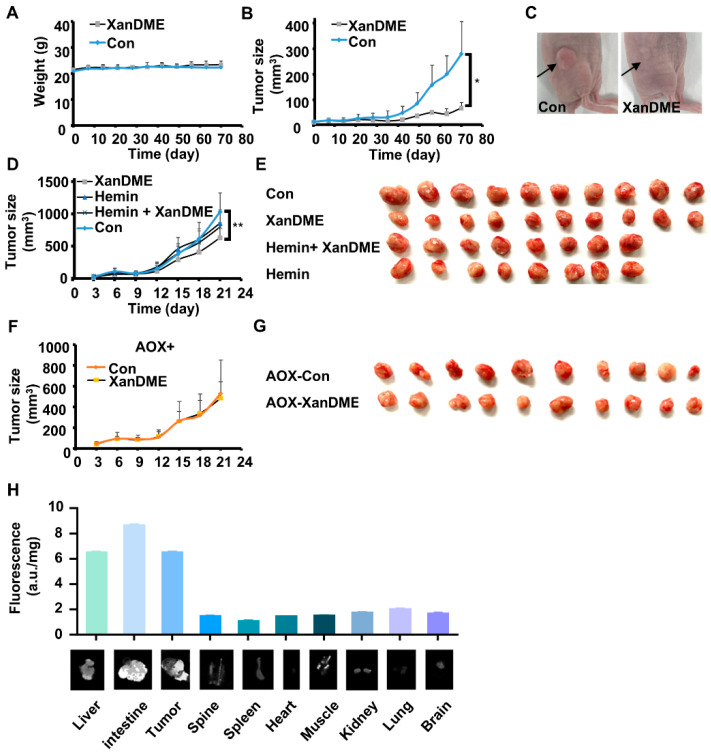
XanDME inhibits TNBC tumor growth. (**A**) The weight of MDA-MB-231 tumor-bearing mice during XanDME treatment (5 mg/kg) via i.p. injection every two days (mean ± SEM, n = 6 independent animals). (**B**) Tumor growth curve of the MDA-MB-231 xenografts. Mice received XanDME (5 mg/kg) via i.p. injection every two days (mean ± SEM, n = 6 independent animals). (**C**) Representative images of the MDA-MB-231 xenografts in the control and XanDME treatment groups, respectively. (**D**) Tumor growth curve of the 4T1 orthotopic allografts in different groups (control and XanDME: n = 10 independent animals; hemin and hemin+ XanDME: n = 8 independent animals). Mice received PBS (Con), XanDME (2.5 mg/kg, i.p. injection daily), hemin (40 mg/kg, i.p. injection every two days), and a combination of XanDME (2.5 mg/kg, i.p. injection daily) and hemin (40 mg/kg, i.p. injection every two days). (**E**) Images of the tumors in different groups of 4T1 tumor-bearing mice at the endpoint. (**F**) Tumor growth curve of the 4T1 (AOX+) orthotopic allografts in different groups (n = 10 independent animals). Mice received either PBS (Con) or XanDME (2.5 mg/kg) via i.p. injection daily. (**G**) Images of the tumors in different groups of 4T1 (AOX+) tumor-bearing mice at endpoint. (**H**) Fluorescence values of XanDME in the various tissues of 4T1 tumor-bearing BALB/c mice with treatment of XanDME (2.5 mg/kg) via i.p. injection daily (n = 10 in each group) (* *p* < 0.05; ** *p* < 0.01).

In order to evaluate the in vivo anti-TNBC efficacy of XanDME, MDA-MB-231 cells were subcutaneously injected into nude mice. Following the establishment of tumors, the mice were randomly divided into two groups, receiving XanDME and PBS, respectively. The overall health and body weight of the mice in both groups were comparable (Figure 5A). Notably, the growth of tumors was significantly impeded in the XanDME group compared to the control group (Figure 5B,C).

We further investigated the impact of XanDME on the orthotopic xenograft model using 4T1 cells. While the effect was not as pronounced as in the MDA-MB-231 model, XanDME significantly delayed tumor growth, an effect could be reversed by co-administration with hemin (Figure 5D,E). Consistent with the in vitro findings (Figure 3D and Figure 4G), 4T1 (AOX+) tumors exhibited a loss of sensitivity to XanDME (Figure 5F,G). These in vivo experiments further confirmed the targeting of XanDME to heme and mitochondrial respiration. At the end of the experiment, the organs of mice bearing 4T1 tumors in the XanDME group were harvested. Tissues were visualized for XanDME using an image scanner, followed by quantification with fluorescence measurements based on XanDME autofluorescence. The results indicated that, in addition to the liver and intestine, XanDME was also highly concentrated in the tumor tissues, underscoring its promising pharmacokinetic profile (Figure 5H).

## 3. Discussion

While mitochondria are emerging targets for certain types of cancer, most efforts have been focused on inhibitors of complex I. However, so far none of these have successfully reached the market. For instance, despite metformin being well documented for being repurposed as an anti-tumor agent, its limited potency hinders clinical application [2]. BAY87-2243 did not progress beyond Phase I clinical trials due to patient intolerance, despite demonstrating potent anti-tumor activity in preclinical studies [33]. More recently, ACS-010759, another specific inhibitor of the mitochondrial complex I, was discontinued during Phase I studies for acute myeloid leukemia and solid tumors due to its narrow therapeutic index [34]. These setbacks have cast a shadow on the prospects of mitochondrial complex I inhibitors and have sparked interest in exploring agents targeting less-studied complexes. Heme-containing cytochromes play a vital role in the functioning of the ETC complexes II, III, and IV. The depletion of heme inhibits their activity, contributing to the anti-AML effect [27]. Therefore, XanDME is promising as an anti-TNBC agent through its binding and depletion of heme.

The interaction between XanDME and heme is not yet fully understood. It has been suggested that Xan and its derivatives could bind to the iron in heme [15]. This hypothesis is supported by our observations of the corresponding peaks from the HRMS analysis. Intriguingly, the mass spectrometry results also suggest a potential hydrolysis of XanDME, as inferred from the observed peaks corresponding to the hydrolyzed XanDME, including [M+H]^+^, [M+Na]^+^, and [M+K]^+^. Furthermore, other newly observed peaks imply that the hydrolyzed XanDME might react with hemin through esterification to form a new compound. Further studies are needed to substantiate these preliminary findings.

Heme, an essential cofactor within cells, remains an underexplored target for drug development. This unique characteristic positions compounds like XanDME and other heme chelators as specific candidates for cancer treatment. However, it is essential to recognize that beyond mitochondrial cytochromes, a diverse array of heme-containing proteins intricately regulate various biochemical processes within cells [35]. Among these hemoproteins, some play pivotal roles in tumorigenesis, cell proliferation, angiogenesis, anti-tumor immune responses, and chemosensitivity. For example, heme mediates the dimerization of progesterone-receptor membrane component 1 (PGRMC1)-influenced interactions with epidermal growth factor receptor (EGFR) and cytochromes P450, impacting cell proliferation and chemoresistance, respectively [36]. The interplay between heme and p53 leads to nuclear export and degradation of the tumor suppressor, ultimately contributing to tumorigenesis [37]. Additionally, the transcription factor BTB and CNC homology1 (BACH1) orchestrates cell metabolism, favoring cancer metastasis and chemoresistance [38,39]. Therefore, targeting BACH1 has emerged as a promising strategy for cancer treatment. However, the intricate reciprocal regulation between heme and BACH1, particularly in the context of XanDME treatment, necessitates further elucidation.

The isocyanide group in XanDME exhibits both nucleophilic and electrophilic characteristics, which enhances its affinity for transition metals [40]. Previous research by Zhu and colleagues demonstrated that isonitrile compounds can inhibit bacterial growth by binding to copper, thereby deactivating key enzymes that rely on copper as cofactor [41]. Despite recent findings suggesting that XanDME has weaker interactions with copper compared to Xan [15], it is crucial to determine whether this copper binding contributes to XanDME’s anti-proliferative effects on TNBC cells. This is particularly relevant given the importance of copper in the functioning of cytochrome c oxidase, also known as mitochondrial complex IV.

Our findings indicate that XanDME not only showed potential in treating TNBC, but also exhibited good druggability. Animal studies revealed that mice treated with XanDME maintained stable health and body weight, comparable to control groups (Figure 5A). This aligns with earlier studies that reported minimal toxicity of XanDME towards primary chick embryo fibroblast cells (CEF), both in the mature and developing stages [13]. A significant finding was the concentration of XanDME within tumor tissues (Figure 5H), possibly due to its lipophilic nature and the lipid-rich structure of mammary tissue. Interestingly, XanDME was found to accumulate most in the gastrointestinal tract, suggesting potential applications in treating gastrointestinal cancers, a prospect that warrants further exploration.

## 4. Materials and Methods

### 4.1. Reagents

The following reagents were used in this study: 5-aminolevulinic acid hydrochloride (5-ALA hydrochloride), XanDME, hemin, and reduced L-glutathione (GSH) were obtained from MCE (Monmouth Junction, NJ, USA). The Annexin V-FITC Apoptosis Detection Kit I was obtained from BD Bioscience (San Jose, CA, USA). The chemical 5-(and-6)-chloromethyl-20,70-dichlorodihydrofluorescein diacetate (CM-H2DCFDA) was obtained from Invitrogen (Carlsbad, CA, USA). Antimycin A was obtained from Abcam (Cambridge, MA, USA). Trichloroacetic acid (TCA), crystal violet, sulforhodamine B (SRB), methanol, formic acid, acetonitrile, oligomycin, rotenone, carbonyl cyanide phospho-(p)-trifluoromethoxy phenylhydrazone (FCCP), sodium pyruvate, uridine, dimethyl sulfoxide (DMSO), sodium azide (NaN_3_), malonate (malonic acid), antimycin A, leflunomide, L-glutamic acid (L-glutamate), L-dihydroorotic acid (DHO), L-glutamine, L-malic acid (malate), 5,5-dithiobis (2-nitrobenzoic acid) (DTNB), decylubiquinone, oxaloacetic acid, potassium ferricyanide, ubiquinone, and 2,6-dichloroindophenol sodium salt hydrate (DCIP) were obtained from Sigma Aldrich (St. Louis, MO, USA). Galactose and glucose were purchased from Psaitong (Beijing, China). HEPES was purchased from Aladdin (Shanghai, China). Fetal bovine serum (FBS), Dulbecco’s modified Eagle’s medium (DMEM) without glucose, and RPMI-1640 medium were obtained from Gibco (Grand Island, NY, USA). The CellTiter-Glo^TM^ Luminescent Cell Viability Assay Kit was obtained from Promega (Madison, WI, USA).

### 4.2. Cell Culture

The cell lines MDA-MB-231, MDA-MB-468, MDA-MB-453, ZR-75-1, 4T1 and MCF10A were cultured in RPMI 1640 medium. The HCT116 cell line was cultured in DMEM medium. These cell lines were obtained from the American Type Culture Collection (ATCC, Manassas, VA, USA). HCT116 SCO2 KO cell line was kindly gifted by Dr. Paul M. Hwang from the National Institutes of Health (NHLBI/NIH, Bethesda, MD, USA). The media was supplemented with 10% fetal bovine serum and 1% Pen Strep Glutamine (100×, 10,000 units/mL penicillin, 10,000 μg/mL streptomycin). The cells were maintained at 37 °C with 5% CO_2_ in a humidified incubator.

### 4.3. AOX Overexpression

The AOX gene was cloned into the pLenti-CMV-AOX-GFP-Puro plasmid and co-transfected with the MISSION packaging mixtures (Sigma Aldrich, St. Louis, MO, USA) into 293T cells. The transfection was performed using the FuGENE HD transfection reagent (Promega, Madison, WI, USA), following the manufacturer’s protocol. After 48 h, the lentivirus supernatant was collected and used to infect the target cells (MDA-MB-468, 4T1). Following an incubation for 72 h, puromycin (2 μg/mL) was added to screen for 4T1 (AOX+) and MDA-MB-468 (AOX+) cells.

### 4.4. In Vitro Cell Proliferation Assay

As previously described, the SRB colorimetric assay was performed to assess the anti-proliferative effects of XanDME on various cancer cell lines [42]. Cells were cultured in 96-well plates at a density of 5 × 10^3^ cells per well. XanDME was added at the indicated concentrations and incubated for the specified time at 37 °C. The cultures were fixed with cold 10% (*w*/*v*) TCA for 1 h at 4 °C and then stained for 10 m with 0.4% (*w*/*v*) SRB. The protein-bound dye was extracted with a 10 mM Tris base solution (pH 10.5), and the absorbance was measured at 515 nm using the SpectraMax 190 microplate reader (Molecular Devices, Sunnyvale, CA, USA). The IC_50_ value was defined as the concentration required for a 50% reduction in cell growth. The relative cell growth rate was determined using the following equation: Relative Growth (%) = OD (treated)/OD (control).

### 4.5. Colony Formation Assay

Cells were seeded in 6-well plates at a density of 2000 cells/well. After the treatments with XanDME for 10 d, the resulting colonies were stained with 0.2% (*w*/*v*) crystal violet in buffered formalin for 20 m. The images of colonies were taken by the Gel Doc XR+ Imaging System (Bio-Rad, Hercules, CA, USA).

### 4.6. Intracellular Regulatory Heme Measurement

Cells from the 4T1 or MDA-MB-231 cell lines were seeded in 24-well plates at a density of 5 × 10^4^ cells/well. The cells were treated with XanDME (5 μM) for 48 h, with or without a 2 h pretreatment with hemin (10 μM). The cells were harvested and lysed with 100 μL of Hemin Assay Buffer from the Hemin Colorimetric Assay Kit (BioVision, Milpitas, CA, USA). The intracellular regulatory heme levels were measured following the manufacturer’s protocol and normalized to the protein content.

### 4.7. Hemin-Promoted Peroxidase Activity Measurement

The Hemin Colorimetric Assay Kit provides a peroxidase activity assay in the presence of hemin, which causes the conversion of a colorless probe to a strongly colored compound (λem = 570 nm). Hemin (5 nM), with or without preincubation with XanDME (25 μM), was added to promote the reaction according to the manufacturer’s protocol.

### 4.8. PPIX Measurement

MDA-MB-231 cells, pretreated with ALA (1 mM) for 2 h, were incubated with XanDME (5 µM) or DMSO for 24 h. A total of 5 × 10^6^ cells were harvested and resuspended in 5 mL EtOAc/acetic acid (3:1, *v*/*v*), and then lysed by sonication (3 × 25 s, 75% intensity; Sonopuls HD 2070 ultrasonic rod, Bandelin Electronic GmbH) with cooling breaks on ice. Cell debris was removed by centrifugation (18,000× *g*, 20 m, 4 °C), and the supernatant was transferred to a new Eppendorf tube. The organic phase was washed twice with H_2_O (1 mL), transferred to a new Eppendorf tube, and 3 M HCl (100 µL) was added to water-solubilize the porphyrins. To quantify the porphyrins, 100 µL of the aqueous phase were transferred into a black flat-bottom 96-well plate, and the fluorescence spectra (λex = 406 nm, λem = 610 nm) were recorded with an Infinite^®^ M200 Pro microplate reader (Tecan, Zürich, Switzerland). The experiment was performed six independent times.

### 4.9. Total Hemin Measurement

MDA-MB-231 cells (1 × 10^5^ cells) were treated with XanDME (5 μM) for 24 h, with or without hemin (10 μM) pretreatment for 2 h. Harvested cells were centrifuged at 300× *g* for 5 m. After removal of the supernatants, 0.5 mL of 2 M oxalic acid solution was added and mixed thoroughly. Sample tubes were put in a heating block set at 100 °C for 30 m. Both blank and sample tubes contained tissue and oxalic acid, but the blanks were not heated (checked for the presence of endogenous porphyrins in the cell). Samples were cooled to room temperature for fluorescent measurements. The fluorescence of porphyrins was read using 400 nm excitation and 662 nm emission. Blank values (parallel unheated samples in oxalic acid) were subtracted from samples, and the fluorescence was normalized with the protein content or cell number.

### 4.10. Intracellular ATP Measurement

MDA-MB-231 cells were cultured in 96-well plates at a density of 1 × 10^4^ cells per well, in either glucose or galactose medium. Cellular ATP levels were assessed using the CellTiter-Glo^TM^ Luminescent Cell Viability Assay Kit from Promega (Madison, WI, USA), following the manufacturer’s protocol, after a 48 h treatment with XanDME (5 μM) with or without a 2 h pretreatment with hemin (10 μM). Furthermore, the luminescence signal was detected using the SpectraMax paradigm microplate reader from Molecular Devices (Sunnyvale, CA, USA). The cellular ATP content was normalized to the cell number.

### 4.11. Fluorescence Measurement of XanDME

A stock solution of XanDME was prepared in acetone to a concentration of 1 mM. Hemin solution was freshly prepared in 0.1 N NaOH to a concentration of 1 mM. XanDME (10 µM), hemin (10 µM), and mixtures of XanDME and hemin in a range of proportions were prepared in DMSO/200 mM HEPES (1:1, *v*/*v*). The assay was conducted in a black-bottom 96-well plate from Corning (Corning, NY, USA) with a final volume of 200 µL. The fluorescence spectra were recorded (λex = 360 nm) using the SpectraMax iD5 Multi-Mode Microplate Reader from Molecular Devices (Sunnyvale, CA, USA).

### 4.12. UV-Vis Spectroscopy

Solutions of XanDME, hemin, and mixtures of XanDME and hemin were prepared as mentioned above. The assay was conducted in a transparent 96-well plate from Thermo Fisher Scientific (Waltham, MA, USA) with a final volume of 200 µL. The UV–Vis spectra were recorded using the SpectraMax iD5 Multi-Mode Microplate Reader.

### 4.13. Seahorse XFp Respirometry Assay

The oxygen consumption rate (OCR) and extracellular acidification rate (ECAR) of MDA-MB-231 cells were assessed using an XFp Analyzer (Seahorse Bioscience, Boston, MA, USA) following the manufacturer’s guidelines. Initially, the cartridge was loaded with XF Calibrant and incubated overnight at 37 °C in a non-CO_2_ environment. MDA-MB-231 cells were then plated in a miniplate with culture medium, containing or lacking XanDME (4 µM), and were cultured at 37 °C in a 5% CO_2_ environment for 48 h. Subsequently, the cells were transferred to unbuffered RPMI 1640 (supplemented with 25 mM glucose and 2 mM L-glutamine, without sodium bicarbonate and phenol red) and incubated at 37 °C in a non-CO_2_ incubator for 1 h. After obtaining six baseline measurements, specific concentrations of 5 μM oligomycin, 3 μM FCCP, 2 μM rotenone, and 0.5 μM antimycin A were subsequently injected, and three measurements were taken. OCR and ECAR were then normalized and calculated using Wave 2.4 software (Seahorse Bioscience, MA, USA) based on the cell numbers.

### 4.14. Mitochondria Isolation from Cells

MDA-MB-231 cells were seeded in three 10 cm dishes. The cells were then scraped up with a rubber spatula in cell culture medium and centrifuged at 300× *g* for 10 m. Following this, the cells were rinsed with 10 mL of cold PBS. The entire cell pellet was resuspended in 1 mL of cold homogenization buffer (0.25 M sucrose, pH 7.4 10 mM HEPES, 1 mM EGTA, 0.1% fatty acid-free BSA), and the cell suspension was transferred to a 1 mL Wheaton Dounce Homogenizer. A tight Teflon pestle was used to stroke up and down 40 times to homogenize the cells on ice. The homogenate was then transferred to a 2 mL Eppendorf tube and centrifuged at 700× *g* for 15 m at 4 °C. The supernatant was transferred to a 1.6 mL Eppendorf tube and centrifuged at 10,000× *g* for 15 min at 4 °C. The pellet was then washed and resuspended in 1 mL cold HB to obtain the mitochondrial fraction.

### 4.15. Mitochondria Isolation from Mouse Liver

Before liver isolation, mice were subjected to a 12 h fasting period, followed by PBS perfusion. The liver tissue (~0.65 g) was then finely chopped and resuspended in 10 mL STE buffer (0.25 M Sucrose, 5 mM Tris-HCl, pH 7.4, 2 mM EGTA, pH~7.4). The tissue was transferred to a chilled 50 mL conical tube. Homogenization was performed with a Glas-Col device set at motor speed of 1600 rpm. This process involved 4 passes, ensuring the Teflon pestle reached the bottom, until a uniform solution was achieved. The homogenate was then centrifuged at 1000× *g* for 5 m at 4 °C in a 50 mL conical tube using a Sigma fixed-angle rotor. After two rounds of washing, the supernatant was centrifuged at 12,000× *g* for 10 m in a pre-cooled Oakridge tube. Finally, the pellet was resuspended in 600 μL of STE buffer.

### 4.16. BN-PAGE for the Complex IV Activity Measurement

Mitochondria (50 μg) were mixed with 20 μL of sample buffer and gently solubilized using a pipette. The mixture was then incubated on ice for 20 m. Following incubation, it was centrifuged at 20,000× *g* for 10 m at 4 °C, and 15 μL of the supernatant was transferred into new tubes. To this supernatant, 2 μL of Coomassie G-250 sample additive was added. An electrophoresis system (XCell SureLock Mini-Cell) was prepared, with a NativePAGE 3–12% gradient gel inserted. For the cathode buffer, Coomassie Brilliant Blue G-250 (0.022 g) was dissolved in 200 mL of Native PAGE anode buffer and mixed thoroughly. The sample (15 μL) of the was loaded into the gel using Prot/Elec Tips. The inner chamber was filled with approximately 180 mL of cathode buffer, and the outer chamber with about 600 mL of running buffer. The BN-PAGE was run at 150 V for 30 m. After 30 m, the cathode buffer was replaced with a clear cathode/running buffer to avoid excessive blue color interference. The gel was then run for an additional 150 m at 250 V to enhance separation. After the run, the gel was carefully removed and placed in ice-cold water. For complex IV activity assessment, the gel was incubated in 20 mL phosphate buffer (pH 7.4) containing diaminobenzidine (0.5 mg/mL) and cytochrome c (1 mg/mL). The appearance of brown bands indicated complex IV activity, with 30–40 m being sufficient for 50 μg of liver protein. The reaction was stopped with 10% acetic acid, followed by washing the gel with water and scanning.

### 4.17. Oxygen Consumption Rate Analysis of Cells and Isolated Mitochondria

Cells were treated with XanDME (5 μM) for 24 h, with or without pretreatment of hemin (10 μM) for 2 h. Isolated mitochondria, at a concentration of 10 mg/mL, were incubated with 30 μM XanDME, with or without the addition of 30 μM hemin for 30 m. Approximately 1 × 10^7^/mL cells in or 1 mg/ mL mitochondria were then transferred into an oxygen consumption chamber (Instech Laboratories, Plymouth Meeting, PA, USA). This was performed in either complete medium or in the respiration buffer (120 mM KCI, 5 mM KH_2_PO_4_, 3 mM HEPES, 1 mM EGTA, 0.3% defatted BSA, 5 mM glutamate, 2.5 mM malate, 0.2 mM ADP, pH~7.4) at a temperature of 37 °C for the cells and isolated mitochondria, respectively. Following the transfer, the chamber was immediately sealed and the oxygen concentration was recorded. The oxygen consumption rate (OCR) was reported as %O_2_ per minute and was normalized to the number of cells or the mass of mitochondria.

### 4.18. Spectrophotometric Measurement of Mitochondrial Complex IV Activity

The activity of mitochondrial complex IV was assessed in accordance with the protocol outlined by Spinazzi et al. [43]. Briefly, isolated mitochondria were resuspended in 10 mM ice-cold hypotonic Tris buffer (pH 7.6) to prepare the mitochondrial extracts. These extracts were then mixed with NaN_3_ (5 mM), the complex IV inhibitor, or XanDME (100 μM) in the reaction buffer (50 mM KH_2_PO_4_/K_2_HPO_4_, pH 7.5). The mixture was incubated at 37 °C for 5 m. The reaction was initiated by the addition of reduced Cytc (60 μM), prepared as previously described [43]. The mixture was immediately analyzed using the SpectraMax iD5 Multi-Mode Microplate Reader (Molecular Devices, Sunnyvale, CA, USA). Measurements were taken every 15 s for 3 m at 550 nm to monitor the rate of reduced Cytc disappearance.

### 4.19. High-Resolution Mass Spectrometry

High-resolution mass spectrometry (HRMS) data were obtained using a SHIMADZU LC–MS 2020 with electrospray ionization (ESI). HRMS data were acquired using a quadrupole-orbitrap (Q-Exactive) mass spectrometer (Thermo Fisher Scientific, Waltham, MA, USA) 10 equipped with a heated electrospray ionization source (HESI-II). The HPLC analysis was performed on an Agilent Technologies (Santa Clara, CA, USA) 1100 Series HPLC system.

### 4.20. Metabolomics Analysis

The MDA-MB-231 cells were treated with either XanDME (5 μM) or DMSO for 48 h. Following this, approximately 107 cells were harvested and washed with PBS. The extraction of cell metabolites and the subsequent metabolomics analysis was conducted as previously described [44].

### 4.21. Hemin Degradation Assay

The GSH-mediated degradation of hemin was conducted based on the method proposed by Ines Hübner et al. [15]. The assay was performed in a transparent Nunc 96-well flat-bottom plate (Thermo Fisher Scientific, Waltham, MA, USA), with a final volume of 200 µL. Stock solutions were prepared with 1 mM XanDME in acetone, 1 mM hemin in 0.1 N NaOH, and 200 mM GSH in H_2_O. Initially, a mixture of hemin (20 μM) and XanDME was incubated at various concentrations at 70 °C for 25 m. Subsequently, GSH (100 mM) was added to initiate the degradation process, excluding the control group, for an additional 10 m. The absorption of hemin was then measured at 400 nm using a SpectraMax iD5 Multi-Mode Microplate Reader (Molecular Devices, Sunnyvale, CA, USA).

### 4.22. Flow Cytometry Analysis of Intracellular XanDME

MDA-MB-231, MDA-MB-468, and 4T1 cells were seeded at 4 × 10^5^/well in 6-well plates. Following a 24 h treatment with XanDME (5, 10, 20 μM), the cells were harvested. The fluorescence of the XanDME within the cells was then measured using the PB450 channel in flow cytometry (Beckman Coulter, Miami, FL, USA).

### 4.23. Fluorescence Microscopy

MDA-MB-231 cells were seeded at 2 × 10^5^/well in 6 well plates. Following a 24 h treatment with XanDME (20 μM), the cells were harvested. Subsequently, the cells were stained with Mitotracker Deep Red (M22426, Thermo Fisher) following the manufacturer’s instruction. Images were captured using a STELLARIS Laser Scanning Confocal Microscopy with a 63×/oil immersion lens from Leica (Wetzlar, Germany).

### 4.24. Xenograft Assay

MDA-MB-231 cells resuspended in PBS (2 × 10^6^/100 μL) were injected subcutaneously into both hind limbs of 4–6 weeks old female nude mice. When tumors reached approximately 50 mm^3^, mice carrying similar tumor burdens were randomized into the XanDME and the control groups (n = 6 in each group). XanDME was administrated by intraperitoneal injection at a dose of 2.5 mg/kg. Mice were monitored for tumor growth every 7 days afterwards. Tumor size was measured using a caliper and calculated using the formula: volume = (length)(width)^2^/2. All procedures were carried out in accordance with guidelines by the Division of Animal Control and Inspection from the Department of Food and Animal Inspection and Control of Macau and were approved by the Animal Care and Use Committee at the Macau University of Science and Technology.

### 4.25. Orthotopic Allograft Assay

Approximately 1 × 10^5^ 4T1 cells in 100 µL of PBS were injected orthotopically into the mammary fat pads of 8-week-old syngeneic female Balb/c mice. When tumors reached approximately 50 mm^3^, mice carrying similar tumor burdens were randomized into different groups. XanDME was administrated by intraperitoneal injection at a dose of 2.5 mg/kg. Mice were monitored for tumor growth every 3 days afterwards. Tumor size was measured using a caliper and calculated using the formula: volume = (length)(width)^2^/2.

Upon reaching the experimental endpoint, the mice were anesthetized, and the tumors were excised. The tissues were then wrapped in plastic wrap and scanned using the Odyssey Image Scanner (Li-COR, Lincoln, NE, USA) under automatic exposure settings. Following the scanning process, the tissues were weighed, and PBS was added in a weight-proportional manner at a concentration of 1 g/mL. The tissues were then homogenized and centrifuged at 700× *g* for 10 m. The supernatant was collected, and the fluorescence intensity was measured (λex = 370 nm, λem = 450 nm) using the SpectraMax iD5 Multi-Mode Microplate Reader, with three replicates for each group.

### 4.26. Statistical Analysis

Data are shown as the mean ± SD (Figure 2, Figure 3 and Figure 4) or mean ± SEM (Figure 5), and the statistical significance was determined by two-tailed Student’s *t*-test or one-way analysis of variance (ANOVA); *p* values less than 0.05 were considered significant (* *p* < 0.05; ** *p* < 0.01; *** *p* < 0.001). The number of biological replicates was listed in each figure.

## 5. Conclusions

In our study, we demonstrated that xanthocillin X dimethyl ether (XanDME) holds significant potential as a therapeutic candidate for triple-negative breast cancer (TNBC). Our findings indicate that XanDME exerts its anti-proliferative effects by binding to heme, which leads to the depletion of intracellular regulatory heme and subsequent mitochondrial dysfunction. This mechanism is evidenced by the disruption of the mitochondrial electron transport chain (ETC), the inhibition of mitochondrial complexes, and the inactivation of mitochondrial respiration.

We highlight the importance of targeting heme as a novel therapeutic strategy for TNBC, given its critical role in mitochondrial function and cancer metabolism. Our results suggest that XanDME could be further explored for its therapeutic applications, particularly in cancers with elevated mitochondrial oxidative phosphorylation (OXPHOS) capacity. Future research should focus on elucidating the detailed molecular mechanisms underlying the interaction between XanDME and heme, as well as exploring the potential of XanDME in preclinical and clinical settings.

In conclusion, our study provides a new perspective on the therapeutic potential of XanDME for TNBC and underscores the importance of targeting mitochondrial heme metabolism as a promising strategy for cancer treatment.

## Data Availability

The authors declare that all data related to this study are available within the article or from the corresponding authors upon request.

## Abbreviations

The following abbreviations are used in this manuscript:

AOX	Alternative Oxidase
ATP	Adenosine Triphosphate
BR	Basal Respiration
CM-H2DCFDA	5-(and-6)-chloromethyl-2′,7′-dichlorodihydrofluorescein diacetate
DMEM	Dulbecco’s Modified Eagle’s Medium
DMSO	Dimethyl Sulfoxide
ECAR	Extracellular Acidification Rate
ETC	Electron Transport Chain
FCCP	Carbonyl Cyanide Phospho-(p)-Trifluoromethoxy Phenylhydrazone
GSH	L-Glutathione Reduced
HRMS	High-Resolution Mass Spectrometry
IC_50_i.p.	Half-Maximal Inhibitory ConcentrationIntraperitoneal Injection
LC–MS	Liquid Chromatography–Mass Spectrometry
MRC	Maximal Respiratory Capacity
OCR	Oxygen Consumption Rate
OXPHOS	Oxidative Phosphorylation
PPIX	Protoporphyrin IX
RH	Regulatory Heme
SRB	Sulforhodamine B
SRC	Spare Respiratory Capacity
TNBC	Triple-Negative Breast Cancer
XanDME	Xanthocillin X Dimethyl Ether

## References

[1] Bianchini G., De Angelis C., Licata L., Gianni L. (2022). Treatment landscape of triple-negative breast cancer—Expanded options, evolving needs. Nat. Rev. Clin. Oncol..

[2] Vasan K., Werner M., Chandel N.S. (2020). Mitochondrial Metabolism as a Target for Cancer Therapy. Cell Metab..

[3] Mahendralingam M.J., Kim H., McCloskey C.W., Aliar K., Casey A.E., Tharmapalan P., Pellacani D., Ignatchenko V., Garcia-Valero M., Palomero L. (2021). Mammary epithelial cells have lineage-rooted metabolic identities. Nat. Metab..

[4] Gong Y., Ji P., Yang Y.-S., Xie S., Yu T.-J., Xiao Y., Jin M.-L., Ma D., Guo L.-W., Pei Y.-C. (2021). Metabolic-Pathway-Based Subtyping of Triple-Negative Breast Cancer Reveals Potential Therapeutic Targets. Cell Metab..

[5] Dutt S., Hamza I., Bartnikas T.B. (2022). Molecular Mechanisms of Iron and Heme Metabolism. Annu. Rev. Nutr..

[6] Pérez-Mejías G., Guerra-Castellano A., Díaz-Quintana A., De la Rosa M.A., Díaz-Moreno I. (2019). Cytochrome c: Surfing Off of the Mitochondrial Membrane on the Tops of Complexes III and IV. Comput. Struct. Biotechnol. J..

[7] Hooda J., Cadinu D., Alam M., Shah A., Cao T.M., Sullivan L.A., Brekken R., Zhang L. (2013). Enhanced heme function and mitochondrial respiration promote the progression of lung cancer cells. PLoS ONE.

[8] Fukuda Y., Wang Y., Lian S., Lynch J., Nagai S., Fanshawe B., Kandilci A., Janke L.J., Neale G., Fan Y. (2017). Upregulated heme biosynthesis, an exploitable vulnerability in MYCN-driven leukemogenesis. JCI Insight.

[9] Sohoni S., Ghosh P., Wang T., Kalainayakan S.P., Vidal C., Dey S., Konduri P.C., Zhang L. (2019). Elevated Heme Synthesis and Uptake Underpin Intensified Oxidative Metabolism and Tumorigenic Functions in Non-Small Cell Lung Cancer Cells. Cancer Res..

[10] Vandekeere S., Dubois C., Kalucka J., Sullivan M.R., García-Caballero M., Goveia J., Chen R., Diehl F.F., Bar-Lev L., Souffreau J. (2018). Serine synthesis via PHGDH is essential for heme production in endothelial cells. Cell Metab..

[11] Canesin G., Di Ruscio A., Li M., Ummarino S., Hedblom A., Choudhury R., Krzyzanowska A., Csizmadia E., Palominos M., Stiehm A. (2020). Scavenging of Labile Heme by Hemopexin Is a Key Checkpoint in Cancer Growth and Metastases. Cell Rep..

[12] Takatsuki A., Suzuki S., Ando K., Tamura G., Arima K. (1968). New antiviral antibiotics; xanthocillin X mono- and dimethylether, and methoxy-xanthocillin X dimethylether. I. Isolation and characterization. (Studies on antiviral and antitumor antibiotics. V). J. Antibiot. (Tokyo).

[13] Takatsuki A., Tamura G., Arima K. (1968). New antiviral antibiotics; xanthocillin X mono- and dimethylether, and methoxy-xanthocillin X dimethylether. II. Biological aspects of antiviral activity. (Studies on antiviral and antitumor antibiotics. VI). J. Antibiot. (Tokyo).

[14] Harms H., Kehraus S., Nesaei-Mosaferan D., Hufendieck P., Meijer L., König G.M. (2015). Aβ-42 lowering agents from the marine-derived fungus *Dichotomomyces cejpii*. Steroids.

[15] Hübner I., Shapiro J.A., Hoßmann J., Drechsel J., Hacker S.M., Rather P.N., Pieper D.H., Wuest W.M., Sieber S.A. (2021). Broad Spectrum Antibiotic Xanthocillin X Effectively Kills *Acinetobacter baumannii* via Dysregulation of Heme Biosynthesis. ACS Central Sci..

[16] Yamaguchi T., Miyake Y., Miyamura A., Ishiwata N., Tatsuta K. (2006). Structure-activity Relationships of Xanthocillin Derivatives as Thrombopoietin Receptor Agonist. J. Antibiot..

[17] Qi-yang H. (2010). Mechanism of inhibiting proliferation by xanthocillin X dimethyl in tumor cells. Chin. J. New Drugs.

[18] Huang L.-H., Xu M.-Y., Li H.-J., Li J.-Q., Chen Y.-X., Ma W.-Z., Li Y.-P., Xu J., Yang D.-P., Lan W.-J. (2017). Amino Acid-Directed Strategy for Inducing the Marine-Derived Fungus *Scedosporium apiospermum* F41–1 to Maximize Alkaloid Diversity. Org. Lett..

[19] Ali M.A., Khan A.U., Ali A., Khaliq M., Khan N., Mujahid S., Calina D., Püsküllüoğlu M., Sharifi-Rad J. (2025). Didemnins as marine-derived anticancer agents: Mechanistic insights and clinical potential. Med. Oncol..

[20] Tamzi N.N., Rahman M., Das S. (2024). Recent Advances in Marine-Derived Bioactives Towards Cancer Therapy. Int. J. Transl. Med..

[21] El-Seedi H.R., Refaey M.S., Elias N., El-Mallah M.F., Albaqami F.M.K., Dergaa I., Du M., Salem M.F., Tahir H.E., Dagliaa M. (2025). Marine natural products as a source of novel anticancer drugs: An updated review (2019–2023). Nat. Prod. Bioprospecting.

[22] Yamamoto T., Orii Y. (1973). Location of heme *a* in cytochrome *a*. I. Combination of alkyl isonitriles with cytochrome *a*. J. Biochem..

[23] Reisberg P., Olson J. (1980). Equilibrium binding of alkyl isocyanides to human hemoglobin. J. Biol. Chem..

[24] Atamna H. (1995). and H. Ginsburg, Heme degradation in the presence of glutathione. A proposed mechanism to account for the high levels of non-heme iron found in the membranes of hemoglobinopathic red blood cells. J. Biol. Chem..

[25] Atamna H., Brahmbhatt M., Atamna W., Shanower G.A., Dhahbi J.M. (2015). ApoHRP-based assay to measure intracellular regulatory heme. Metallomics.

[26] Hakkaart G.A.J., Dassa E.P., Jacobs H.T., Rustin P. (2005). Allotopic expression of a mitochondrial alternative oxidase confers cyanide resistance to human cell respiration. EMBO Rep..

[27] Lin K.H., Xie A., Rutter J.C., Ahn Y.-R., Lloyd-Cowden J.M., Nichols A.G., Soderquist R.S., Koves T.R., Muoio D.M., MacIver N.J. (2019). Systematic Dissection of the Metabolic-Apoptotic Interface in AML Reveals Heme Biosynthesis to Be a Regulator of Drug Sensitivity. Cell Metab..

[28] Orlicka-Płocka M., Gurda-Wozna D., Fedoruk-Wyszomirska A., Wyszko E. (2020). Circumventing the Crabtree effect: Forcing oxidative phosphorylation (OXPHOS) via galactose medium increases sensitivity of HepG2 cells to the purine derivative kinetin riboside. Apoptosis.

[29] Sullivan L.B., Gui D.Y., Hosios A.M., Bush L.N., Freinkman E., Vander Heiden M.G. (2015). Supporting Aspartate Biosynthesis Is an Essential Function of Respiration in Proliferating Cells. Cell.

[30] Van Vranken J.G., Rutter J. (2015). You Down With ETC? Yeah, You Know D!. Cell.

[31] Martínez-Reyes I., Cardona L.R., Kong H., Vasan K., McElroy G.S., Werner M., Kihshen H., Reczek C.R., Weinberg S.E., Gao P. (2020). Mitochondrial ubiquinol oxidation is necessary for tumour growth. Nature.

[32] Huang J., Du J., Lin W., Long Z., Zhang N., Huang X., Xie Y., Liu L., Ma W. (2019). Regulation of lactate production through p53/β-enolase axis contributes to statin-associated muscle symptoms. EBioMedicine.

[33] Ellinghaus P., Heisler I., Unterschemmann K., Haerter M., Beck H., Greschat S., Ehrmann A., Summer H., Flamme I., Oehme F. (2013). BAY 87-2243, a highly potent and selective inhibitor of hypoxia-induced gene activation has antitumor activities by inhibition of mitochondrial complex I. Cancer Med..

[34] Yap T.A., Daver N., Mahendra M., Zhang J., Kamiya-Matsuoka C., Meric-Bernstam F., Kantarjian H.M., Ravandi F., Collins M.E., Di Francesco M.E. (2023). Complex I inhibitor of oxidative phosphorylation in advanced solid tumors and acute myeloid leukemia: Phase I trials. Nat. Med..

[35] Wang T., Ashrafi A., Modareszadeh P., Deese A.R., Castro M.D.C.C., Alemi P.S., Zhang L. (2021). An Analysis of the Multifaceted Roles of Heme in the Pathogenesis of Cancer and Related Diseases. Cancers.

[36] Kabe Y., Nakane T., Koike I., Yamamoto T., Sugiura Y., Harada E., Sugase K., Shimamura T., Ohmura M., Muraoka K. (2016). Haem-dependent dimerization of PGRMC1/Sigma-2 receptor facilitates cancer proliferation and chemoresistance. Nat. Commun..

[37] Shen J., Sheng X., Chang Z., Wu Q., Wang S., Xuan Z., Li D., Wu Y., Shang Y., Kong X. (2014). Iron metabolism regulates p53 signaling through direct heme-p53 interaction and modulation of p53 localization, stability, and function. Cell Rep..

[38] Wiel C., Le Gal K., Ibrahim M.X., Jahangir C.A., Kashif M., Yao H., Ziegler D.V., Xu X., Ghosh T., Mondal T. (2019). BACH1 Stabilization by Antioxidants Stimulates Lung Cancer Metastasis. Cell.

[39] Lee J., Yesilkanal A.E., Wynne J.P., Frankenberger C., Liu J., Yan J., Elbaz M., Rabe D.C., Rustandy F.D., Tiwari P. (2019). Effective breast cancer combination therapy targeting BACH1 and mitochondrial metabolism. Nature.

[40] Emsermann J., Kauhl U., Opatz T. (2016). Marine Isonitriles and Their Related Compounds. Mar. Drugs.

[41] Zhu M., Wang L., Zhang W., Liu Z., Ali M., Imtiaz M., He J. (2020). Diisonitrile-Mediated Reactive Oxygen Species Accumulation Leads to Bacterial Growth Inhibition. J. Nat. Prod..

[42] Huang X.-M., Huang J.-J., Du J.-J., Zhang N., Long Z., Yang Y., Zhong F.-F., Zheng B.-W., Shen Y.-F., Huang Z. (2021). Autophagy inhibitors increase the susceptibility of KRAS-mutant human colorectal cancer cells to a combined treatment of 2-deoxy-D-glucose and lovastatin. Acta Pharmacol. Sin..

[43] Spinazzi M., Casarin A., Pertegato V., Salviati L., Angelini C. (2012). Assessment of mitochondrial respiratory chain enzymatic activities on tissues and cultured cells. Nat. Protoc..

[44] Huang J., Long Z., Lin W., Liao X., Xie Y., Liu L., Ma W. (2018). Integrative omics analysis of p53-dependent regulation of metabolism. FEBS Lett..

